# Loss of WWOX contributes to cisplatin resistance in triple-negative breast cancer cells by modulating miR-182 and miR-214

**DOI:** 10.55730/1300-0144.5891

**Published:** 2024-07-02

**Authors:** Bahadır BATAR, Elif SERDAL, Berna ERDAL, Hasan OĞUL

**Affiliations:** 1 Department of Medical Biology, Faculty of Medicine, Tekirdağ Namık Kemal University, Tekirdağ, Turkiye; 2 Department of Tumor Biology and Immunology, Institute of Health Sciences, Tekirdağ Namık Kemal University, Tekirdağ, Turkiye; 3 Department of Medical Microbiology, Faculty of Medicine, Tekirdağ Namık Kemal University, Tekirdağ, Turkiye; 4 Department of Computer Sciences and Communication, Faculty of Computer Sciences, Østfold University College, Halden, Norway

**Keywords:** Cisplatin resistance, microRNA, triple-negative breast cancer, WWOX

## Abstract

**Background/aim:**

WW domain-containing oxidoreductase (WWOX) loss frequently occurs in triple-negative breast cancer (TNBC). WWOX loss enhances cisplatin resistance in TNBC patients. Although WWOX loss has an effect on the selection of a DNA repair pathway that contributes to enhanced mutagenesis, the downstream expression changes in resistant cancer cells have not been fully explored. This study aimed to investigate the potential role of microRNAs (miRNAs) in the regulation of cisplatin resistance in WWOX-deficient TNBC cells.

**Materials and methods:**

Transient transfections were performed to overexpress WWOX in MDA-MB-231 cells. WWOX-overexpressing MDA-MB-231 cells were determined by western blot. Expression profiling of the miRNA was assessed via real-time polymerase chain reaction.

**Results:**

miRNA expression profiling of WWOX-deficient and -sufficient MDA-MB-231 cells revealed that miR-182 upregulation and miR-214 downregulation were markedly positively associated with cisplatin resistance of WWOX-deficient MDA-MB-231 cells. An elevated expression of miR-182 and decreased expression of miR-214 may contribute to cisplatin resistance in WWOX-absent MDA-MB-231 cells by signaling pathway dysregulation of DNA repair/apoptosis/ protein kinase B (AKT).

**Conclusion:**

The results emphasize that WWOX deficiency promotes resistance to cisplatin in TNBC cells and the possible predicting biomarker of WWOX for resistance to cisplatin.

## 1. Introduction

The WW domain-containing oxidoreductase (WWOX) gene is located on chromosome 16 and encompasses a common chromosomal fragile region, FRA16D. This region is the most active fragile locus in many epithelial human cancers [1].

The WWOX gene encodes a tumor suppressor protein with two N-terminal WW (conserved tryptophan residues) domains involved in protein-protein interactions and a short-chain dehydrogenase (SDR) domain that plays a role in steroid metabolism [1].

Allele loss at the WWOX locus has most commonly been observed in hormonal-regulated cancers such as breast, ovarian, and prostate cancers [2]. Although there has been information reported on the relationship between WWOX protein deficiency and cancer development [3], the tumor suppressor function of WWOX has not yet been fully elucidated.

Breast cancer is the most commonly diagnosed cancer among women. It leads to high mortality due to the metastasis of cancer cells to distant organs. Surgical resection, neoadjuvant, and adjuvant chemotherapy are common treatment approaches for breast cancer. Furthermore, a combination of chemotherapy with hormonal therapy and HER2-targeted treatment has improved the survival of breast cancer patients [4]. Drug resistance to chemotherapy in triple-negative breast cancer (TNBC) is the main obstacle to the efficacy of cancer therapies. Therefore, there is an urgent need to develop new strategies in reversing chemotherapy resistance of TNBC cells.

Cisplatin is among the most effective wide-spectrum platinum-based anticancer agents commonly used to treat multiple malignant tumors. Cisplatin resistance is a basic issue in the treatment of breast cancer and causes treatment failure in metastatic cancer patients [5]. The cytotoxic effect of cisplatin in normal tissues and cisplatin resistance gained by cancer cells decrease the clinical efficacy of this drug [6]. Increased DNA repair or apoptosis signaling pathway defects are resistance development mechanisms. However, the mechanisms of cisplatin resistance have not been fully defined.

Various epigenetic and posttranscriptional changes may lead to the development of drug resistance by affecting DNA repair mechanisms, the apoptosis signaling pathway, expression of drug transporters, cell cycle checkpoints, or tumor microenvironment.

MicroRNAs (miRNAs) posttranscriptionally regulate gene expression. miRNAs are small noncoding RNA molecules. More than 30 miRNAs have been indicated to regulate platinum susceptibility in many tumors [7].

Our previous findings first demonstrated that WWOX loss or reduction significantly contributes to cisplatin resistance of TNBC cells and provides a survival advantage to cancer cells. A key role seems to be exerted by WWOX deficiency in the resistance of TNBC cells to cisplatin treatment. Although it is known that WWOX loss promotes cisplatin resistance through effects on the choice of a DNA repair pathway that contributes to enhanced mutagenesis [8], the downstream expression changes in resistant cancer cells have not been fully explored. This study aimed to investigate the possible effects of miRNAs in the regulation of resistance to cisplatin in WWOX-negative TNBC cells.

## 2. Materials and methods

### 2.1. Cell culture, chemicals, and treatments

MDA-MB-231 cell lines were kindly gifted by Prof. Figen Celep Eyupoglu (Karadeniz Technical University, Trabzon, Türkiye). MDA-MB-231 cells are cisplatin-resistant TNBC cells. They were grown in Dulbecco’s modified eagle’s medium (Gibco; Thermo Fisher Scientific Inc., Waltham, MA, USA) supplemented with 10% fetal bovine serum and 100 μg/mL gentamicin. Cisplatin (MedChemExpress LLC, Monmouth Junction, NJ, USA; HY-17394), was dissolved in dimethylsulfoxide (DMSO), and stored as a stock solution at −20 °C until use. The MDA-MB-231 cells were exposed to 100 μM of cisplatin for 4 h [9].

### 2.2. Transformation

pCMV-WWOX recombinant plasmid and pCMV-empty vector (pCMV-EV) were kindly gifted by Prof. Kay Huebner (Ohio State University, Columbus, OH USA). Propagation of the plasmids was performed in One Shot TOP10 Chemically Competent *E. coli* (Invitrogen; Thermo Fisher Scientific Inc.) according to the manufacturers’ instructions. Briefly, 2 μL of pCMV-WWOX or pCMV-EV was directly added onto the competent cells. Each transformation vial was spread on Lennox L agar (LB agar Invitrogen; Thermo Fisher Scientific Inc.) plates supplemented with ampicillin (35 μg mL^−1^, Gibco; Thermo Fisher Scientific Inc.). Then, colonies were selected for plasmid isolation.

### 2.3. Bacteria culture conditions and plasmid DNA isolation

Each transformed bacteria colony was grown in liquid Luria-Bertoni broth (LB, Invitrogen; Thermo Fisher Scientific Inc.) medium supplemented with 35 μg mL^−1^ of ampicillin at 37°C, overnight. Plasmid DNA isolation was performed using PureLink Quick Plasmid Miniprep Kit (Invitrogen’ Thermo Fisher Scientific Inc.) according to the manufacturer’s instructions.

### 2.4. Transient transfections

pCMV-WWOX recombinant and pCMV-EV plasmid DNAs were separately transfected into the MDA-MB-231 cells using Lipofectamine 2000 (Invitrogen; Thermo Fisher Scientific Inc.) according to the transfection reagent instructions. The transfected cells were incubated at 37 °C and 5% CO_2_ for 48 h and then the WWOX protein expression was detected via western blot analysis.

### 2.5. Western blot

Cell lysis was carried out in radioimmunoprecipitation assay buffer (Thermo Fisher Scientific Inc.) supplemented with Halt Protease cocktail inhibitors (Thermo Fisher Scientific Inc.). Proteins were immunoblotted with anti-WWOX polyclonal antibody (Abclonal, 1:1000), antibeta actin polyclonal antibody (Invitrogen, 1:1000; Thermo Fisher Scientific Inc.), and goat antirabbit IgG (H+L) secondary antibody, horseradish peroxidase (Invitrogen, 1:3000; Thermo Fisher Scientific Inc.).

### 2.6. miRNA extraction and cDNA synthesis

The MDA-MB-231 TNBC cells were transiently transfected with pCMV-WWOX or pCMV-EV. Forty-eight hours after the transfection, the cells were exposed to cisplatin (100 μM) for 4 h [9]. Following transfection and cisplatin treatment, total RNAs, including miRNAs, were extracted from the MDA-MB-231 cells using a MiRNeasy Mini Kit (Qiagen GmbH, Hilden, Germany) according to manufacturers’ instructions. A miCURY LNA RT Kit (Qiagen GmbH) was used to synthesize first-strand cDNA from the total RNAs according to the manufacturer’s instructions.

### 2.7. Quantitative real-time polymerase chain reaction (qPCR)

qPCR was performed using the miRCURY LNA miRNome Human PCR Panel (Qiagen GmbH) with 372 LNA PCR assays for the amplification of human miRNAs, three interplate calibrators, 3 LNA PCR assays for reference genes, five RNA spike-in control PCR assays, and one blank well. The miRNA target sequences (LNA-enhanced primers) for human miRNAs were predefined by the supplier. Reactions were performed on a LightCycler 480 system (Roche Diagnostics, Basel, Basel-Stadt, Switzerland). The second derivative method was used to analyze the initial data.

### 2.8. Statistical analysis

Functional analysis of miRNAs requires the integration of several data from different information sources. Herein, a miRNA set enrichment analysis (miSEA) tool [10] supporting the integration of experimental data and based on biological literature knowledge to conduct new hypotheses was used. The miSEA follows the original procedure of the Gene Set Enrichment Analysis (GSEA) platform [11] for miRNA. A list of the differentially expressed miRNAs and their values for the miSEA algorithm is given below:

1. Ranking of the differentially expressed miRNAs
2. For each miRNA set:
  2.1. Compute the cumulative sum of the ranked miRNAs.
  2.2. Record the enrichment score (ES) as the maximum deviation from zero.
  2.3. Report normalized ES (NES).
3. Report miRNA sets ranked by their NES values.

Step 2.1 begins with the initialization of the sum, then goes through the ranked list of miRNA to enhance the sum if the current miRNA is within the target group and reduce it if it is not. miSEA calculates the NES to determine the degree to which the current miRNA set is overrepresented in the resulting differential expression profile. The p-value refers to the significance of the corresponding NES calculation.

The miRNA sets are classified into six categories in the miSEA: target, regulator, cluster, family, tissue, and function. The regulator categories of the miRNA sets were assembled from the TransmiR web server [12]. The target categories of the miRNA sets were created according to their common target genes. Two experimentally validated databases, miRTarBase [13] and miRecords [14], retrieved the relationships between the miRNA and target in an updated version of the miSEA. The cluster category contains miRNA sets including miRNAs with adjacent genomic coordinates. miRNA sets of cluster categories were added from the TAM server. The design of the family category is such that miRNAs considered to have occurred from duplicated copies of common ancestor miRNAs are grouped into the same miRNA sets. The classifications of the miRNA family were collected from miRBase [15]. The tissue category requires tissue-specific microRNA sets produced by collecting miRNAs with an index of tissue specificity values ≥0.7 from the DIANA MicroT server [16]. Many of the function categories of miRNA sets were collected from the TAM server. Other sets obtained from a recent study suggest regulating the protein network in the EGFR-driven cell cycle by cocoordination of some microRNAs [17].

Target predictions were performed using TargetScan [18], microRNA.org [19], MicroCosm [20], Diana [16,] and miRDB [21]. miRNA that were predicted as regulators of WWOX by at least two tools were further included in the analysis.

## 3. Results

**Figure 1:**
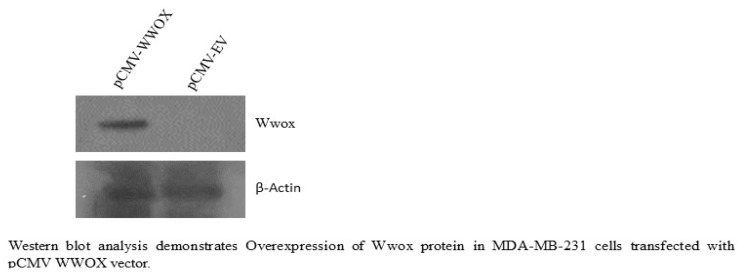
Overexpression of WWOX in MDA-MB-231 cells.

WWOX was overexpressed in the MDA-MB-231 cells (Figure 1). When invoked between two experimental conditions (WWOX-sufficient or -deficient), the miSEA revealed that 144 miRNA sets were enriched, which fell into six clusters, eight families, 40 functions, 22 regulators, 68 targets and three tissues. Six miRNA sets were statistically significant at p ≤ 0.05 (Table 1).

Four functional sets that were believed to be important pathways for cisplatin resistance, apoptosis, DNA repair, cell proliferation, and protein kinase B (AKT) pathways were further analyzed. All these annotations were included in the miSEA output list with an ES higher than the required threshold (0.25 in absolute) (Table 2).

The list of miRNAs that were predicted to be potential regulators of WWOX is shown in Table 3.

According to these metaanalyses, Table 4 shows miRNAs that were found to be related to the expression of WWOX under cisplatin treatment.

**Figure 2:**
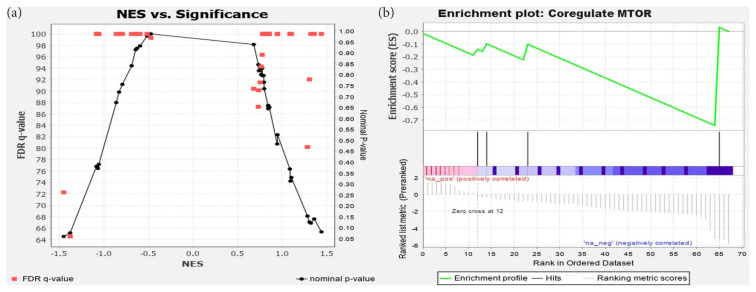
miSEAs in the function category: (a) significance plot of all the miRNA sets in the category, and (b) plot of their ESs.

The significance analysis specific miRNA set, Coregulated MTOR, which involves hsa-mir-182, is shown in Figure 2. Figure 2a plots the miRNA sets in the function category by their NES vs. the nominal p-value (shown in black circles) and NES vs. the false discovery rate (FDR, shown in red squares). As shown, the lowest FDR was achieved with the second highest absolute NES at p = 0.05. This set, which corresponds to miRNAs regulated by MTOR was the only miRNA set significantly enriched in this category. The plot of the ES for this miRNA set is discerned in Figure 2b. This plot visualizes the same miRNA sets by their ES (not normalized) vs. their rank in the same category from positively correlated to negative correlated, where Coregulated MTOR attained the second rank in the negatively correlated end. These plots justify both empirically and statistically that coregulation of MTOR was a significant factor in the experiment.

**Figure 3:**
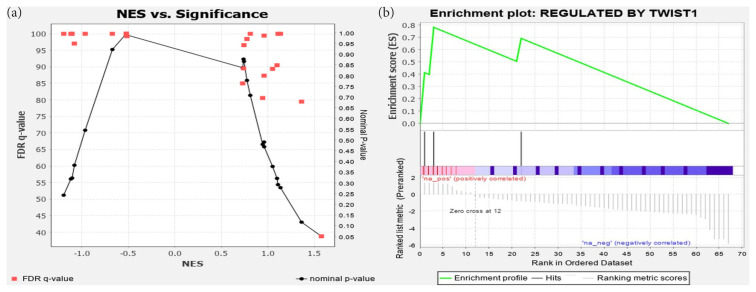
miSEAs in the regulator category: (a) significance plot of all the miRNA sets in the category, and (b) plot of their ESs.

Similar results can be observed for the miRNA set Regulated by TWIST1, which involves hsa-mir-214 (Figure 3). Figure 3a plots the miRNA sets in the regulator category by their NES vs. the nominal p-value (shown in black circles) and NES vs. the FDR (shown in red squares). It shows that the lowest FDR was achieved with the highest absolute NES at p = 0.05. This set, which corresponds to Regulated by TWIST1, was the only miRNA set significantly enriched in this category. Figure 3b discerns the plot of the ES for this miRNA.

## 4. Discussion

Currently, chemotherapy is a commonly used treatment approach for breast cancer patients. Although breast cancer patients initially respond well to chemotherapy, drug resistance remains one of the major obstacles to treatment failure, particularly for invasive and metastatic breast cancer [5,6]. Comprehensive studies have been performed to identify the molecular mechanisms involved in the cisplatin-based chemotherapy resistance of TNBC cells. However, pathological processes and molecular mechanisms underlying the cisplatin sensitivity of TNBC cells are still not completely understood and studies are ongoing to prevent or overcome cancer resistance to cisplatin treatment. Therefore, the identification of cisplatin sensitivity would develop new therapeutic strategies to overcome cisplatin resistance in TNBC patients.

In a previous study, we examined the possible mechanisms enabling cisplatin treatment resistance. The principal finding of that initial study suggested that reduced WWOX expression, which commonly occurs in cancers, results in resistance to cisplatin [8]. The findings were consistent with the data from different types of cancer studies. For example, WWOX significantly sensitized cisplatin-treated osteosarcoma Saos-2 cells to undergo cell death/apoptosis [22]. WWOX-expressing ovarian cancer stem cells showed more sensitivity to cisplatin treatment [23]. These results demonstrated that WWOX might be responsible for the cisplatin sensitivity of cancer cells.

Dysregulated miRNA expression levels are well known in many cancer types and are potentially significant players in regulation of drug resistance and sensitivity.

One study demonstrated the association of WWOX with an expression of miRNAs in TNBC cells. Khaweled et al. [24] suggested that WWOX enhances the expression of miR-146a causing reduced invasion and metastasis in TNBC cells. In the other type of cancer study, Ekizoglu et al. [25] showed that reduced WWOX expression has been associated with miR-134 expression in head and neck squamous cell carcinoma (HNSCC).

Herein, it was aimed to evaluate the role of miRNAs in the modulation of WWOX-deficient TNBC cells resistance to cisplatin. Using the MDA-MB-231 TNBC cell line as a main in vitro cell model, miRNA expression profiling was performed in both WWOX-deficient (empty vector-transfected, pCMV-EV) and -sufficient (WWOX-transfected, pCMV-WWOX) human MDA-MB-231 cells. Based on the functional miRNA sets’ enrichment miSEA and metaanalyses, two selected candidate miRNAs (miR-182 and miR-214) were identified to potentially contribute to breast cancer cell resistance to cisplatin in WWOX-deficient MDA-MB-231 cells. It was found that miR-182 and miR-214 were dysregulated in the WWOX-deficient MDA-MB-231 cells in comparison to the WWOX-sufficient MDA-MB-231 cells. The miR-182 expression was significantly upregulated in the WWOX-deficient MDA-MB-231 cells. Moreover, the expression of miR-214 was markedly downregulated in the WWOX-deficient MDA-MB-231 cells. According to the metaanalyses, WWOX is a predictive target of miR-182. Herein, novel data were reported on the cisplatin resistance modulated by miR-182 and miR-214 due to WWOX deficiency in MDA-MB-231 cells. miR-182 and miR-214 could be promising therapeutic targets for cisplatin resistance in WWOX-deficient TNBC cells.

The major effect of cisplatin is interfering with DNA repair and accelerating cell apoptosis by inducing DNA damage in various types of cancers. Enhanced DNA repair activity and reduced apoptosis induction are the primary aberrant DNA damage response mechanisms for cancer resistance to cisplatin [26]. As shown in the miSEA, the upregulation of miR-182 was able to enhance cisplatin resistance of the TNBC cells via targeting DNA repair and/or apoptosis pathways. Moreover, the analysis suggested that the downregulation of miR-214 could result in cisplatin resistance due to aberrant apoptosis and AKT signaling pathways, respectively.

Currently, many studies have focused on the role of miR-182 in response to cisplatin treatment in various cancers, and have revealed especially increased expression of miR-182 in cisplatin-resistant cancers. Seidl et al. [27] showed that miR-182-5p overexpression significantly enhanced the sensitivity of cisplatin-resistant lung adenocarcinoma cells. Another study found a markedly upregulation of miR-182 expression in cisplatin-resistant nonsmall cell lung cancer (NSCLC) A549 cells [28]. Consistent with these results, Ning et al. [29] revealed that increased expression of miR-182 was related to the resistance of NSCLC cells to cisplatin by downregulating programmed cell death 4 (PDCD4). In addition to these results, Qin et al. [30] demonstrated that miR-182 expression levels were significantly enhanced in cisplatin-resistant human liver cancer (HepG2/R) cells than in HepG2 cells. miR-182 increased cisplatin resistance of hepatocellular carcinoma cells by targeting tumor protein 53-induced nuclear protein1 (TP53INP1). According to these data, miR-182 functions as an onco-miR in many cancers. The current study also revealed that miR-182 is an onco-miR in WWOX-deficient TNBC and that miR-182 may be a potential therapeutic candidate and biomarker in WWOX-deficient TNBC therapy.

Many experiments have been performed to compare the expression pattern of miR-214 between cisplatin-resistant and sensitive cancer cells. Recently, Wang et al. revealed that miR-214-3p expression was decreased in cisplatin-resistant oral squamous cell carcinoma (OSCC) cells [31]. Furthermore, they showed that downregulation of miR-214-3p inhibited apoptosis in cisplatin-resistant OSCC cells.

To date, there are no data on the role of tumor suppressor WWOX on cisplatin resistance by modulating miRNA expression in cancer cells.

## 5. Conclusions

The data herein suggested that loss of WWOX expression leads to significantly increased expression of miR-182 and decreased expression of miR-214 associated with TNBC cell resistance to cisplatin. We propose that the loss of WWOX expression in TNBC contributes to deregulation of the DNA repair and apoptosis pathways via modulation of the miR-182 and miR-214 expression.

## Figures and Tables

**Table 1:** Enriched miRNA sets between two conditions.

miRNA set	Enrichment Score	p-value	miRNAs included
miR-506 Family	−0.89	0.05	hsa-mir-512, hsa-mir-509
Coregulate MTOR	−0.74	0.05	hsa-mir-99a, hsa-mir-100, hsa-mir-182, hsa-mir-199b
Regulated by TWIST1	0.78	0.05	hsa-mir-214, hsa-mir-200b, hsa-mir-205
Regulate FGA, FGB, FGG	0.91	0.03	hsa-mir-409, hsa-mir-29c
Regulate MUC17	0.85	0.05	hsa-mir-30c, hsa-mir-20a
Regulate APP	0.85	0.05	hsa-let-7d, hsa-mir-20a

**Table 2:** Manually selected functional miRNA sets enriched in miSEA analysis.

miRNA set	Enrichment score	p-value	miRNAs included
Apoptosis	−0.25	0.05	hsa-mir-182, hsa-mir-34a, hsa-mir-210
AKT Pathway	0.36	0.05	hsa-mir-195, hsa-mir-29c, hsa-mir-20a, hsa-mir-182, hsa-mir-15b, hsa-mir-34a, hsa-let-7g, hsa-mir-210
Cell proliferation	0.46	0.05	hsa-mir-214, hsa-mir-20a, hsa-mir-205, hsa-mir-331
DNA Repair	0.48	0.03	hsa-let-7d, hsa-mir-9, hsa-mir-15b

**Table 3:** Predicted miRNAs targeting WWOX.

miRNA	Prediction source
miR-186-5p	TargetScanMouse, microRNA.org
miR-182-5p	TargetScanMouse, microRNA.org
miR-449c-5p	MicroCosm, microRNA.org
miR-495-3p	Diana, MicroCosm
miR-449b	MicroCosm, microRNA.org
miR-200c-3p	Diana, microRNA.org
miR-449a-5p	MicroCosm, microRNA.org
miR-486a-5p	MicroCosm, microRNA.org
miR-429-3p	Diana, microRNA.org
miR-742-3p	Diana, MicroCosm
miR-34c-5p	MicroCosm, microRNA.org
miR-696	MicroCosm, miRDB
miR-34a-5p	MicroCosm, microRNA.org
miR-34b-5p	MicroCosm, microRNA.org
miR-154-5p	miRDB, microRNA.org
miR-466f-5p	MicroCosm, miRDB
miR-200b-3p	Diana, microRNA.org

**Table 4:** Selected miRNAs associated with WWOX expression.

Name	Description
hs-mir-182	• Annotated by enriched sets “Coregulated MTOR, Apoptosis, DNA Repair” WWOX is predicted target
hsa-mir-214	• Annotated by enriched sets “Regulated by TWIST1, AKT Pathway”

## References

[1] Aldaz CM Ferguson BW Abba MC WWOX at the crossroads of cancer, metabolic syndrome related traits and CNS pathologies Biochimica et Biophysica Acta (BBA)-Reviews on Cancer 2014 1846 1 188 200 10.1016/j.bbcan.2014.06.001 24932569 PMC4151823

[2] Karras JR Schrock MS Batar B Huebner K Fragile genes that are frequently altered in cancer: players not passengers Cytogenetic and Genome Research 2017 150 3–4 208 216 10.1159/000455753 28199992

[3] Ludes-Meyers JH Bednarek AK Popescu NC Bedford M Aldaz CM WWOX, the common chromosomal fragile site, FRA16D, cancer gene Cytogenetic and Genome Research 2003 100 1–4 101 110 10.1159/000072844 14526170 PMC4150470

[4] Wang Y Minden A Current molecular combination therapies used for the treatment of breast cancer International Journal of Molecular Sciences 2022 23 19 11046 10.3390/ijms231911046 36232349 PMC9569555

[5] Roy V Pockaj BA Allred JB Apsey H Northfelt DW A Phase II trial of docetaxel and carboplatin administered every 2 weeks as preoperative therapy for stage II or III breast cancer: NCCTG study N0338 American Journal of Clinical Oncology 2013 36 6 10.1097/COC.0b013e318256f619 PMC3493827 22868240

[6] Mayer IA Abramson VG Lehmann BD Pietenpol JA New strategies for triple-negative breast cancer—deciphering the heterogeneity Clinical Cancer Research 2014 20 4 782 790 10.1158/1078-0432.CCR-13-0583 24536073 PMC3962777

[7] Fang C Chen YX Wu NY Yin JY Li XP MiR-488 inhibits proliferation and cisplatin sensibility in non-small-cell lung cancer (NSCLC) cells by activating the eIF3a-mediated NER signaling pathway Scientific Reports 2017 7 1 40384 10.1038/srep40384 28074905 PMC5225486

[8] Schrock MS Batar B Lee J Druck T Ferguson B Wwox–Brca1 interaction: role in DNA repair pathway choice Oncogene 2017 36 16 2215 2227 10.1038/onc.2016.389 27869163 PMC5398941

[9] Cataldo A Cheung DG Balsari A Tagliabue E Coppola V miR-302b enhances breast cancer cell sensitivity to cisplatin by regulating E2F1 and the cellular DNA damage response Oncotarget 2016 7 1 786 797 10.18632/oncotarget.6381 26623722 PMC4808033

[10] Çorapçıoğlu ME Oğul H miSEA: microRNA set enrichment analysis Biosystems 2015 134 37 42 10.1016/j.biosystems.2015.05.004 26093049

[11] Subramanian A Tamayo P Mootha VK Mukherjee S Ebert BL Gene set enrichment analysis: a knowledge-based approach for interpreting genome-wide expression profiles Proceedings of the National Academy of Sciences 2005 102 43 15545 15550 10.1073/pnas.0506580102 PMC1239896 16199517

[12] Wang J Lu M Qiu C Cui Q TransmiR: a transcription factor–microRNA regulation database Nucleic Acids Research 2010 38 suppl_1 119 122 10.1093/nar/gkp803 PMC2808874 19786497

[13] Hsu SD Tseng YT Shrestha S Lin YL Khaleel A miRTarBase update 2014: an information resource for experimentally validated miRNA-target interactions Nucleic Acids Research 2014 42 78 85 10.1093/nar/gkt1266 PMC3965058 24304892

[14] Xiao F Zuo Z Cai G Kang S Gao X miRecords: an integrated resource for microRNA–target interactions Nucleic Acids Research 2009 37 suppl_1 105 110 10.1093/nar/gkn851 PMC2686554 18996891

[15] Kozomara A Griffiths-Jones S miRBase: annotating high confidence microRNAs using deep sequencing data Nucleic Acids Research 2014 42 68 73 10.1093/nar/gkt1181 PMC3965103 24275495

[16] Maragkakis M Reczko M Simossis VA Alexiou P Papadopoulos GL DIANA-microT web server: elucidating microRNA functions through target prediction Nucleic Acids Research 2009 37 suppl_2 273 276 10.1093/nar/gkp292 PMC2703977 19406924

[17] Uhlmann S Mannsperger H Zhang JD Horvat EÁ Schmidt C Global microRNA level regulation of EGFR-driven cell-cycle protein network in breast cancer Molecular Systems Biology 2012 8 1 570 10.1038/msb.2011.100 22333974 PMC3293631

[18] Agarwal V Bell GW Nam JW Bartel DP Predicting effective microRNA target sites in mammalian mRNAs Elife 2015 4 e05005 10.7554/eLife.05005 26267216 PMC4532895

[19] Betel D Wilson M Gabow A Marks DS Sander C The microRNA. org resource: targets and expression Nucleic Acids Research 2008 36 suppl_1 149 153 10.1093/nar/gkm995 PMC2238905 18158296

[20] Kozomara A Birgaoanu M Griffiths-Jones S miRBase: from microRNA sequences to function Nucleic Acids Research 2019 47 155 162 10.1093/nar/gky1141 PMC6323917 30423142

[21] Wong N Wang X miRDB: an online resource for microRNA target prediction and functional annotations Nucleic Acids Research 2015 43 146 152 10.1093/nar/gku1104 PMC4383922 25378301

[22] Salah Z Bar-Mag T Kohn Y Pichiorri F Palumbo T Tumor suppressor WWOX binds to ΔNp63α and sensitizes cancer cells to chemotherapy Cell Death and Disease 2013 4 1 e480 e480 10.1038/cddis.2013.6 23370280 PMC3564006

[23] Yan HC Xu J Fang LS Qiu YY Lin XM Ectopic expression of the WWOX gene suppresses stemness of human ovarian cancer stem cells Oncology Letters 2015 9 4 1614 1620 10.3892/ol.2015.2971 25789010 PMC4356412

[24] Khawaled S Suh SS Abdeen SK Monin J Distefano R WWOX inhibits metastasis of triple-negative breast cancer cells via modulation of miRNAs Cancer Research 2019 79 8 1784 1798 10.1158/0008-5472.CAN-18-0614 30622118

[25] Ekizoglu S Bulut P Karaman E Kilic E Buyru N Epigenetic and genetic alterations affect the WWOX gene in head and neck squamous cell carcinoma PloS One 2015 10 1 e0115353 10.1371/journal.pone.0115353 25612104 PMC4303423

[26] Xiao M Guo J Xie L Yang C Gong L Let-7e suppresses DNA damage repair and sensitizes ovarian cancer to cisplatin through targeting PARP1 Molecular Cancer Research 2020 18 3 436 447 10.1158/1541-7786.MCR-18-1369 31722968

[27] Seidl C Panzitt K Bertsch A Brcic L Schein S MicroRNA-182-5p regulates hedgehog signaling pathway and chemosensitivity of cisplatin-resistant lung adenocarcinoma cells via targeting GLI2 Cancer Letters 2020 469 266 276 10.1016/j.canlet.2019.10.044 31697978

[28] Li W Wang W Ding M Zheng X Ma S MiR-1244 sensitizes the resistance of non-small cell lung cancer A549 cell to cisplatin Cancer Cell International 2016 16 1 1 7 10.1186/s12935-016-0305-6 27073334 PMC4828824

[29] Ning FL Wang F Li ML Yu ZS Hao YZ MicroRNA-182 modulates chemosensitivity of human non-small cell lung cancer to cisplatin by targeting PDCD4 Diagnostic Pathology 2014 9 1 1 5 10.1186/1746-1596-9-143 25012722 PMC4108001

[30] Qin J Luo M Qian H Chen W Upregulated miR-182 increases drug resistance in cisplatin-treated HCC cell by regulating TP53INP1 Gene 2014 538 2 342 347 10.1016/j.gene.2013.12.043 24447717

[31] Wang X Li H Shi J LncRNA HOXA11-AS promotes proliferation and cisplatin resistance of oral squamous cell carcinoma by suppression of miR-214-3p expression BioMed Research International 2019 Article ID 8645153. 10.1155/2019/8645153 PMC6558628 31275988

